# Whole-genome sequences of *Enterobacter cloacae* H5 and *Enterobacter bugandensis* H6 isolated from patients in Khartoum State, Sudan

**DOI:** 10.1186/s12863-026-01471-1

**Published:** 2026-07-10

**Authors:** Hiba A. A. Mahgoub, Mogahid M. Elhassan, Hind H. Ahmed, Hisham N. Altyab, Mohamed E. Hamid

**Affiliations:** 1 Department of Microbiology, Medical Laboratory Sciences Program, Nahrain College, Khartoum, Sudan; 2 Department of Microbiology, Faculty of Medical Laboratory Sciences, National University, Khartoum, Sudan; 3 Department of Microbiology, College of Medical Laboratory Science, Sudan University of Science and Technology, P. O Box 407, Khartoum, Sudan; 4 Biochemistry Department, Faculty of Sciences, King Abdulaziz University, Jeddah, Saudi Arabia; 5 Department of Clinical Microbiology and Parasitology, College of Medicine, King Khalid University, Abha, Saudi Arabia

**Keywords:** MDR *Enterobacter*, Genomic features, Digital DNA-DNA hybridization, AMR genes, Phylogenetic analysis, *Whole-genome sequencing*, *E.cloacae*, *E. bugandensis*

## Abstract

**Objective:**

*Enterobacter* species are important nosocomial pathogens, mainly due to multidrug resistance (MDR). This study reports the whole-genome sequences of *Enterobacter cloacae* H5 and *Enterobacter bugandensis* H6.

**Data description:**

Digital DNA-DNA hybridization (dDDH) confirmed species identity: H5 (91.8% with *E. cloacae* ATCC 13047) and H6 (89.3% with *E. bugandensis* EB-247). H5 has 46 contigs, 4,867,245 bp, GC 54.97%; H6 has 27 contigs, 4,616,569 bp, GC 56.05%. Phylogenetic analysis grouped H5 within *E. cloacae* and H6 within *E. bugandensis*. Both genomes harbor multiple antimicrobial resistance (AMR) genes. H5 includes β-lactamase CMH-16, FosA2, efflux pumps (oqxA, emrB), and regulators (ramA, baeR, baeS). H6 includes β-lactamase ACT-146, FosA2, efflux pumps (oqxA, emrB, msbA), regulators (baeR, baeS), and target modification gene ArnT. These genomes highlight the clinical significance of these strains and provide essential resources for understanding the genomic basis of antimicrobial resistance in Sudanese *Enterobacter* isolates.

**Supplementary Information:**

The online version contains supplementary material available at https://doi.org/10.1186/s12863-026-01471-1.

## Objective

*Enterobacter*, a genus of Gram-negative, facultative anaerobic, rod-shaped bacteria, is recognized as a significant cause of nosocomial infections worldwide [1]. These pathogens can produce a wide range of infections, including bacteremia, respiratory tract infections, and urinary tract infections, particularly in immunocompromised patients [2]. *Enterobacter* species, *for example E. aerogenes*, *E. cloacae*, and *E. hormaechei*, are part of the ESKAPE pathogens, recognized for their multidrug-resistant phenotypes and ability to thrive in hospital environments [3].

The increase of antibiotic resistance in *Enterobacter* has been promoted by the common use of broad-spectrum antibiotics, leading to many outbreaks and difficulties in efficient treatment [4]. These organisms regularly display intrinsic resistance mechanisms, comprising the production of AmpC β-lactamases (Mezzatesta et al., 2012) and extended-spectrum β-lactamases (ESBLs) [1], reducing treatment options.

In spite of extensive research into their resistance profiles, the pathogenic mechanisms of *Enterobacter* remain poorly known [2, 5]. The introduction of genome sequencing technology offers a hopeful means for solving the genetic basis of resistance and virulence [6, 7]. This study aims to investigate whether the whole-genome sequences of Sudanese *Enterobacter* isolates differ from global reference genomes and to identify any novel mutations associated with antimicrobial resistance. Understanding these genetic variants is essential for tackling the public health threat caused by multidrug-resistant *Enterobacter* species in resource-limited settings.

## Data description

This study presents a genomic analysis of *Enterobacter cloacae* strain H5 and *Enterobacter bugandensis* strain H6, both isolated from patients in Khartoum State, Sudan, as part of a surveillance initiative. An overview of all datasets generated in this study is provided in Table 1.

**Table 1:** Overview of datasets generated in this study

Label	Name of data file/data set	File types (file extension)	Data repository and identifier (DOI or accession number)
Dataset 1	*Enterobacter cloacae* H5 RefSeq genome assembly	FASTA (.fna)	NCBI GenBank: https://identifiers.org/insdc:GCF_052615725.1 [8]
Dataset 2	*Enterobacter cloacae* H5 GenBank genome assembly	FASTA (.fna)	NCBI GenBank: https://identifiers.org/insdc:GCA_052615725.1 [9]
Dataset 3	*Enterobacter cloacae* H5 whole genome shotgun project	WGS sequence files	NCBI WGS: https://identifiers.org/insdc:JBQXFY01 [10]
Dataset 4	*Enterobacter bugandensis* H6 RefSeq genome assembly	FASTA (.fna)	NCBI GenBank: https://identifiers.org/insdc:GCF_052615685.1 [11]
Dataset 5	*Enterobacter bugandensis* H6 GenBank genome assembly	FASTA (.fna)	NCBI GenBank: https://identifiers.org/insdc:GCA_052615685.1 [12]
Dataset 6	*Enterobacter bugandensis* H6 whole genome shotgun project	WGS sequence files	NCBI WGS: https://identifiers.org/insdc:JBQXFX01 [13]
Dataset 7	Figure 1.pdf	PDF (.pdf)	https://doi.org/10.5281/zenodo.18639881 [14]
Dataset 8	Supplementary_Dataset_S1_CARD_H5.xlsx	Excel spreadsheet (.xlsx)	https://doi.org/10.5281/zenodo.18639881 [14]
Dataset 9	Supplementary_Dataset_S2_CARD_H6.xlsx	Excel spreadsheet (.xlsx)	https://doi.org/10.5281/zenodo.18639881 [14]
Dataset 10	Table 1. Comparative genomic features of *Enterobacter cloacae* H5 *and Enterobacter bugandensis* H6.pdf	PDF (.pdf)	https://doi.org/10.5281/zenodo.18639881 [14]

Genome assembly statistics, including genome size, GC content, N50, and gene counts of the two strains compared to the type strains are summarized in Supplementary Table S1, which is available in the associated data repository.

**Fig. 1:**
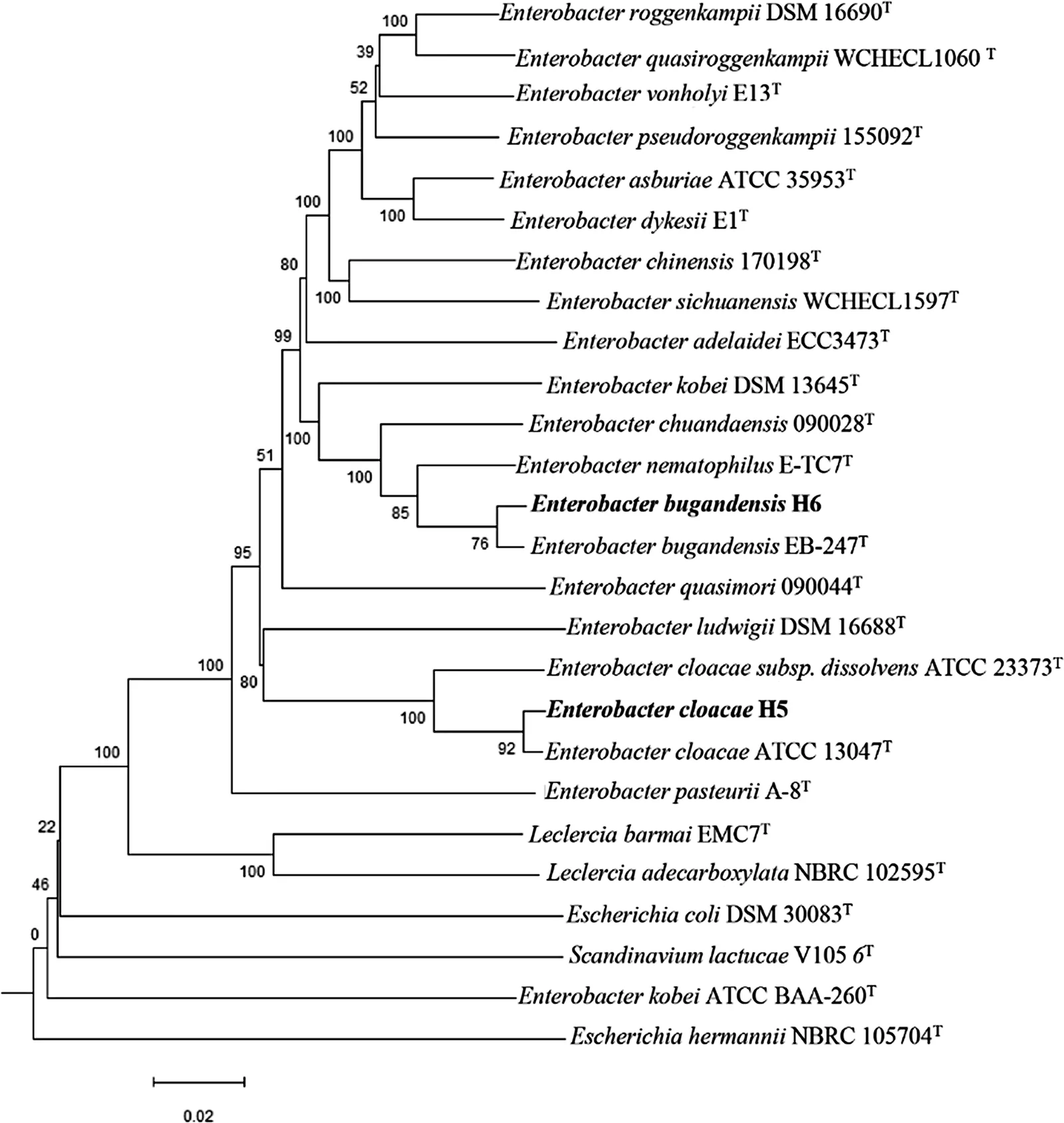
Phylogenomic tree generated by FastME 2.1.6.1 using GBDP distances derived from genome sequences. Branch lengths indicate GBDP distance (d5. Numbers above the branches represent GBDP pseudo-bootstrap support (greater than 60%) from 100 replications, with an average support of 78.1%. The tree is rooted at the midpoint. According to TYGS phylotaxonomic analysis, *Enterobacter cloacae* H5 is classified within *Enterobacter cloacae* ATCC 13047 ^T^, and *Enterobacter bugandensis* H6 is classified within *Enterobacter bugandensis* EB-247^T^

These strains’ taxonomic location was explained by the use of dDDH, which confirmed that they were *E. cloacae* (aligning with *E. cloacae* ATCC 13047) and *E. bugandensis* (aligning with *E. bugandensis* EB-247), respectively (Fig. 1) which is available in the associated data repository. The dDDH values, along with genome sequencing, serve as robust methods for bacterial species delineation [15].

The finding of multiple antimicrobial resistance genes in both genomes is a significant worry, indicating the global increase in antibiotic resistance among *Enterobacteriaceae* [16].

Antimicrobial resistance genes were identified using CARD and RGI under strict criteria. In *E. cloacae* H5, β-lactamase (CMH-16), FosA2, and efflux pump and regulatory genes (oqxA, emrB, ramA, baeR, baeS) were detected. In *E. bugandensis* H6, class C β-lactamase (ACT-146), FosA2, efflux pump and regulatory genes (oqxA, emrB, msbA, baeR, baeS), and ArnT, involved in peptide antibiotic target modification, were identified. All AMR genes for H5 and H6 were summarized in the AMR genes file, available in the associated data repository. The identified resistance mechanisms, including antibiotic efflux pumps, antibiotic inactivation enzymes, and target alterations, highlight the diverse strategies employed by these bacteria to evade the effects of antimicrobial agents. These findings feature the clinical relevance of these strains, especially taking into account the rising prevalence of multidrug-resistant *Enterobacteriaceae* in hospital sites [5].

The study’s findings support existing literature on the role of *Enterobacter* species as opportunistic pathogens capable of causing a range of infections, including bacteremia, lower respiratory tract infections, and urinary tract infections. The presence of multiple antimicrobial resistance genes in these strains agrees with the established capacity of *Enterobacteriaceae* to acquire and spread resistance genes through horizontal gene transfer [16]. This draws attention to the value of continued surveillance and monitoring of antimicrobial resistance patterns in *Enterobacteriaceae* to address appropriate treatment plans and infection control measures [17, 18].

*E. cloacae* and *E. bugandensis* have emerged as clinically significant nosocomial pathogens, predominantly in immunocompromised patients. An *E. cloaca* is known to cause a range of infections, including pneumonia, septicemia, and urinary tract infections, while *E. bugandensis* has been associated with neonatal sepsis [17]. The identification of specific resistance genes, such as beta-lactamase genes (bla genes), colistin-resistant genes, tetracycline-resistant genes, and aminoglycoside-resistant genes, provides valuable information for understanding the mechanisms of resistance in these strains and for developing strategies to combat them [19].

## Limitations

Two isolates from a specific geographic area were the sole focus of the investigation, which might not accurately reflect the genetic diversity of these species [20]. This Data Note describes genome sequences from two clinical isolates obtained from a single geographic location, which may not reflect the broader genomic diversity of *Enterobacter* species circulating in other regions. Although the study found several genes linked to antibiotic resistance, it lacked functional tests to verify the genes’ expression and activity. Phenotypic antimicrobial susceptibility testing data were not available for these isolates; therefore, direct correlations between resistance genotypes and phenotypic resistance profiles could not be assessed. In addition, plasmid localization of resistance genes was not experimentally validated, and resistance determinants were identified based on in silico predictions. However, this limitation does not affect the validity of the genomic data presented, and plasmid localization can be investigated in future studies. The precise role that these genes play in the resistance phenotypes that have been reported requires more investigation. Other elements that might contribute to the virulence and pathogenicity of these strains were not thoroughly examined in the study, which mainly concentrated on genes linked to antibiotic resistance. Future studies integrating phenotypic data, plasmid characterization, and larger isolate collections will further enhance the interpretation of these genomic findings. The entire spectrum of virulence factors and their function in infection require more investigation. Notwithstanding these drawbacks, this research offers insightful information about the genomic traits of *E. cloacae* and *E. bugandensis*.

## Supplementary Information

Below is the link to the electronic supplementary material.

Supplementary Material 1

Supplementary Material 2

Supplementary Material 3

## Data Availability

The whole-genome sequences of Enterobacter cloacae H5 and Enterobacter bugandensis H6 have been deposited in the NCBI GenBank database under accession numbers GCA_052615725.1 (RefSeq: GCF_052615725.1) and GCA_052615685.1 (RefSeq: GCF_052615685.1), respectively. Both genomes are associated with BioProject PRJNA1320714. The corresponding BioSample accession numbers are SAMN51164549 for E. cloacae H5 and SAMN51164550 for E. bugandensis H6. The WGS project accessions are JBQXFY01 and JBQXFX01, respectively.Additionally, all associated data files, including the AMR genes file, tables, and figures, have been deposited in Zenodo and are accessible via DOI: https://doi.org/10.5281/zenodo.18639881.

## Abbreviations

dDDH: Digital DNA-DNA Hybridization
DDH: DNA-DNA Hybridization
DSM: Deutsche Sammlung von Mikroorganismen und Zellkulturen
G + C: Guanine + Cytosine content
GC: Guanine-Cytosine (content)
GGDC: Genome-to-Genome Distance Calculator
ML: Maximum-Likelihood
N50: A metric for genome assembly quality
Qiagen: (Brand name, not an abbreviation)
RAST: Rapid Annotation using Subsystem Technology
SEED: (Refers to the SEED database, not an abbreviation)
TYGS: Type (of) Genome Server
