# Supplementary material for: Whole-genome sequences of Enterobacter cloacae H5 and Enterobacter bugandensis H6 isolated from patients in Khartoum State, Sudan
Source: BMC Genom Data. 2026 Jul 10;27:83. doi: 10.1186/s12863-026-01471-1 (PMC13640266; doi:10.1186/s12863-026-01471-1)
Supplement: Supplementary file 1 [file 12863_2026_1471_MOESM3_ESM.pdf]

Supplementary Table S1. Comparative genomic features of *Enterobacter cloacae* H5 and *Enterobacter bugandensis* H6.

**Attribute**	*Enterobacter cloacae* H5	*Enterobacter cloacae* ATCC 13047^T	*Enterobacter bugandensis* H6	*Enterobacter bugandensis* EB-247^T
**Genome Size (bp)**	4,867,245	5,314,581	4,616,569	4,717,613
**GC Content (%)**	54.97	54.79	56.05	56.00
**N50**	320,568	NA	559,961	NA
**L50**	3	1	3	1
**Number of Subsystems**	381	386	371	373
**Number of Coding Sequences**	4,615	5,244	4,390	4,435
**Number of RNAs**	72	109	70	108
